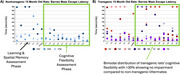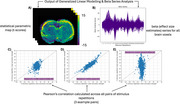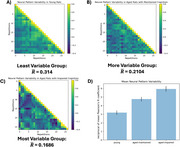# The neurophysiological underpinnings of brain resilience: a BOLD fMRI deep phenotyping study

**DOI:** 10.1002/alz70856_097055

**Published:** 2025-12-24

**Authors:** Emma Pineau, Keying Chen, Margaret M Koletar, Maged Goubran, John G Sled, Bojana Stefanovic

**Affiliations:** ^1^ University of Toronto, Toronto, ON, Canada; ^2^ Sunnybrook Research Institute, Toronto, ON, Canada; ^3^ Department of Medical Biophysics, University of Toronto, Toronto, ON, Canada; ^4^ Mouse Imaging Centre ‐ Hospital for Sick Children, Toronto, ON, Canada

## Abstract

**Background:**

A poorly understood facet of AD is the complex relationship between the burden of pathology and cognition, as evidenced by the phenomenon of cognitive reserve. Recent single cell recording techniques have reported that normal brain function involves continuous remodelling of functional neuronal properties, even under stable environmental conditions, leading to time‐dependent variation in neuronal responses to stimulation, though, to the best of our knowledge, this neuronal variability hasn’t been investigated across a brain‐wide scale. We hypothesize that *microscopic* variability in neuronal activation patterns results in *microscopic* variability in vascular reactivity which manifests as *mesoscopic* variability in blood oxygenation level dependent (BOLD) functional magnetic resonance imaging (fMRI) activation patterns. We further hypothesize that the fMRI activation pattern variability may be a robust neurophysiological correlate of cognitive reserve.

**Methods:**

We have undertaken a protracted BOLD fMRI protocol to deeply characterise individual response variability across repetitive somatosensory stimulations in transgenic Fischer 344 rat model (TGF344‐AD) that expressed the APPswe and PS1ΔE9 mutations, leading to age‐related development amyloid, tau phosphorylation, frank neuronal loss, and cognitive decline. We have determined that 30% of our transgenic animals show little to no cognitive impairment (Figure 1), supporting the use of this model in studying cognitive reserve.

**Results:**

The subjectwise parametric activation maps (Figure 2A) and the corresponding effect‐size (beta) series (Figure 2B) were used to examine trial‐by‐trial voxel‐level response variability. To quantify both temporal and spatial variability, we plotted the variability of all pairwise stimulation trial combinations in Figure 2C‐E and quantified the variability in the activation patterns using a Pearon's correlation. The animals were then categorized into 3 groups: young (Figure 3A), cognitively maintained aged (Figure 3B), and cognitively impaired aged (Figure 3C). Figure 3A‐C shows the average Pearson's correlation coefficient for each pair of stimulus repetitions across all rats in the group. The amount of brain activation pattern variability increased with age and degree of cognitive impairment (Figure 3D).

**Conclusion:**

Our proposed assay is sensitive to group‐wise differences in activation pattern variability that correlates with cognitive performance.